# Interpersonal and communication skills development in dermatology residency: Results of a single-institution needs assessment

**DOI:** 10.1016/j.jdin.2024.07.011

**Published:** 2024-08-16

**Authors:** Elizabeth E. Bailey, Bryan K. Dang, Luqman Mushila Hodgkinson, Rachel Schwartz, Merisa Kline, Barbette Weimer-Elder, Kristin M. Nord

**Affiliations:** a Department of Dermatology, Stanford University School of Medicine, Stanford, California; b Design-Impact Engineering Program, Stanford University D.School, Stanford, California; c Department of Anesthesia and Perioperative Care, University of California, San Francisco, San Francisco, California; d Service Excellence, Stanford Health Care, Stanford, California

**Keywords:** dermatology, interpersonal and communication skills, medical education, milestones, needs assessment

*To the Editor:* Clinician interpersonal and communication skills are increasingly being recognized as a critical component of high-quality patient care and the ACGME Dermatology Milestones 2.0 includes 3 Interpersonal and Communication Skills (ICS) milestones. Studies have identified specialty-specific needs in residency training on these skills but no such studies have been performed in dermatology, a specialty which likely faces unique communication needs due to unusually short visits and the often chronic and incurable nature of skin conditions.^1^ The purpose of this study is to understand dermatology residents’ perspectives on educational content and approaches needed for effective interpersonal and communication skills development.

A total of 14/16 (88%) invited postgraduate year 3 and 4 residents participated in the focus group in July 2019. A written handout was used to guide discussion (see Supplementary Appendix, available via Mendeley at https://doi.org/10.17632/kn8pmt5kk4.1). The focus group, 62 minutes in duration, was conducted by a researcher (B.K.D.), who was not known to the participants and had a nonhierarchical relationship with them. The focus group was audio recorded and transcribed using Otter.ai and manually edited to correct any errors or missing segments. Four authors participated in the coding and analysis: a male medical student (L.M.H.), a male nurse and ed-tech startup founder (B.D.), a female dermatologist and residency program director (E.E.B.), and a female dermatologist and former residency program director (K.M.N.). Two authors (L.M.H. and B.K.D.) coded the transcript independently using a qualitative description approach, followed by an iterative thematic analysis performed by authors E.E.B. and K.M.N., with disagreements resolved by consensus. This project did not meet the definition of human subject research as determined by the Stanford University IRB and did not require IRB review.

Two key themes emerged from the qualitative analysis: (1) educational content needs (Table I), and (2) effective educational approaches (Table II) for interpersonal and communication skills. Five subthemes emerged under the theme of educational content needs and 4 subthemes emerged under effective educational approaches. Residents perceived limitation in adopting approaches taught and role modelled by their faculty when there was a difference in appearance between trainee and supervisor (Table II).

**Table I:** Educational content needs for interpersonal and communication skills development identified by dermatology residents

Subtheme	Representative quotes
**Establishing educational need** Value of demonstrating the need for interpersonal and communication skills development for learners	“What stands out to me is…a 30-something year-old guy with a new diagnosis of cancer and … being in that room and feeling like, ‘Why do we not have a better way of learning, like this is never going to be easy, like this is really, really hard.’ And then … trying to think about that.”
**Building rapport with patients quickly** Identified need for dermatologists to connect and establish rapport with a patient quickly and effectively	“In dermatology, in particular, with a very high volume of patients, you have to be able to connect with someone and communicate very effectively, very efficiently. And I think that's something that's always a struggle, because I think, from my prior experiences in medical school, we had kind of an excess of time with patients … but now you're like starting in biologic and 10 minutes getting out there. So I think that it is a challenge to try and convey a lot of information quickly and to develop rapport. And to do that in a very short amount of time.
**Communicating diagnostic uncertainty** Identified need for providers to know how to communicate diagnostic uncertainty both to other providers and to patients	“I think one thing I would add is how to communicate uncertainty both between providers and your patient? …Do you use numbers, do you use analogies?”
**Communicating between care team members** Identified need to communicate respect between care team members	“The attendings, … when there's a tough day, they'll say to the staff ‘You know, guys, today was a really rough day, and everybody did a really great job.’ And then sometimes they'll even … point out what each person did that was good, I've seen that done and I feel like it's really effective to lift people up after a talk.”
**Adapting communication strategies to situational context** Identified need to understand the role of different communication styles and adapting approach to situation	“Okay, and I think one other thing to point out is just how communication styles are dependent on the situation and the patient. Right? So Dr X works with pediatric patients, anxious parents, she doesn't come in with… bravado.”

**Table II:** Effective educational approaches for interpersonal and communication skills development identified by dermatology residents

Subtheme	Representative quotes
**Value of experiential learning approaches**	“Role-playing and scenarios…I found it to be more memorable than sitting and listening to a lecture.”
**Observation of supervisors** Reflections on learning which has occurred through direct observation of supervisors/role modelling from faculty	[about attending] “it’s just part of her communicating with patients and establishing her rapport. The second that she walks into the room. It’s amazing. And it’s not so much what she says, but her mannerisms. And kind of like the unspoken language, you can be a patient who doesn’t speak English with an interpreter and the second she walks in, she immediately has a connection with them. It’s something that I’ve really strived to adopt as well.”
**Role of direct feedback** Importance of getting direct feedback from those directly observing conversations with patients	“And then the next family meeting, he was like, I’m going to have you run the family meeting and discuss, and I’m going to be a fly on the wall. And I’ll only interject if you get into trouble. And then right after that, he…gave me feedback. And I found that to be really useful, because I kind of watched him do it, then I had to just do it. And then I got immediate feedback on things that he thought were done well, and things he would have done differently.”
**Overcoming barriers to role modeling based on learner experience** A trainee feels that he/she/they is unable to emulate the approach of a supervisor if there is a difference in appearance between resident and supervisor [examples provided included gender identity, age, height] which is a learning barrier that needs to be addressed	“I’m just saying, I think [Attending] is a great communicator, I worked with him for a year, but I could not pull that off…I do not look like him.”

Educational content needs highlighted by participants in this study overlap significantly with those previously identified in other specialties, such as managing time constraints and communicating diagnostic uncertainty.^2^^,^^3^ The effective educational approaches that residents identified in this study are also consistent with those identified as best practices by expert focus groups.^4^ These approaches are also well-aligned to be paired with coaching, which has been used to promote communication skills development in residency training.^5^ Limitations to this needs assessment include lack of generalizability as it was performed at a single institution and the limited scope of this assessment to perspectives of current dermatology residents.

This needs assessment provides insight into dermatology residents’ perspectives on educational content and approaches effective for ICS development. Next steps from this study include evaluating the efficacy of a communication skills curriculum crafted to address the educational content needs identified and teaching approaches including the use of communication coaching to cultivate expertise in ICS competencies for dermatology resident learners.

## Conflicts of interest

None declared.
